# Modified technique in Freyer's prostatectomy to achieve hemostasis

**DOI:** 10.4103/0970-1591.56189

**Published:** 2009

**Authors:** Vinay V. Shahapurkar, Nishant Khare, Avanish V. Deshmukh

**Affiliations:** Department of Surgery, JNMC, DMIMS, Sawangi (Meghe), Wardha, India

**Keywords:** Benign prostatic hyperplasia, blood loss, Freyer's prostatectomy, hemostasis, traction

## Abstract

This study is an attempt to develop a technique by which complete hemostasis can be achieved on table by giving traction to the Foley's catheter thereby compressing the venous plexus and the avulsed prostatic arteries at the bladder neck by the inflated balloon. A total of 170 cases of BPH were operated by Freyer's Suprapubic Trans-vesicle prostatectomy. In the technique, bladder mucosa is reposited below the balloon and the balloon is inflated to 60 ml of normal saline. The balloon is kept at the bladder neck and traction is applied to the catheter. Traction is maintained by strapping the catheter to the thigh of the patient with sticking plaster for 24–48 h. The average blood loss was 18.9 ml which proves that the Foley's balloon pressure traction method at the bladder neck is effective in achieving hemostasis in patients undergoing open prostatectomy.

## INTRODUCTION

Benign prostatic hyperplasia (BPH) is the most common benign tumor in men and its incidence is age related.[1] Surgery for BPH has evolved from the days of perineal approach to the currently popular transurethral resection. Until 20-25 years ago, while open surgery was the most common approach, in the late 1970s, the development of endoscopes gradually reduced open surgical operations.[2] The ratio of open surgery to endoscopic resection has large variations among different countries and even among various areas of a vast country like India. Although TURP is considered as the gold standard, it is still out of reach for a vast majority of rural population due to unavailability of expertise or equipments. The rate of complications has come down heavily but still complete hemostasis remains an enigma. History elucidates the continuous attempts done to achieve complete hemostasis. This is because the prostate is deeply situated in the pelvis and its blood supply comes from the deeper planes. This study is an attempt to develop a technique by which complete haemostasis can be achieved on table by giving traction to the Foley's catheter thereby compressing the venous plexus and the avulsed prostatic arteries at the bladder neck by the inflated balloon. This technique is an innovation in the open prostatectomy which can be widely practiced in the rural areas.

## MATERIALS AND METHODS

The present study was conducted from June 2005 to October 2007, in the patients of Benign prostatic hyperplasia (BPH) admitted in the surgery ward at AVBR hospital, Sawangi, after the approval from the Ethical Committee of the above institute and with legal consent of patients. The procedure was carried out under the supervision of senior Surgeon.

Patient selection: A total of 170 cases of BPH were operated by Freyer's Suprapubic Trans-vesicle prostatectomy. All the patients presented with symptomatic BPH. They were all admitted and treated as in-patients.

Preoperative workup: Detailed clinical history of each patient was recorded and AUA symptom score was calculated. All patients were subjected to thorough clinical examination. Renal function tests, serum electrolytes, Urine culture and antibiotic sensitivity was done for all the patients. X-ray KUB and USG KUB was done and prostate size and post void residual urine was calculated. Serum PSA was done only if Digital Rectal Examination was suspicious.

Surgery: After making the bladder full with normal saline by per urethral catheter, a suprapubic midline incision was taken and abdomen opened in layers. Bladder was opened with a stab knife. Prostate was supported with a finger passed per rectally and enucleation of prostate was done. After enucleation of prostate in addition to the index finger, middle finger was inserted and the rectum was pulled caudally. This helps to exert minimal pressure over the venous plexus. A 22 – 24 No three way foley's catheter is placed in the bladder and the bladder mucosa is reposited below the balloon posteriorly, by the finger. The balloon is inflated to 60 ml of normal saline and is kept at the bladder neck and traction is applied to the catheter. Traction is maintained by strapping the catheter to the thigh of the patient with sticking plaster for 24 to 48 h. On table, clear urine is confirmed and bladder is closed primarily in two layers. No suprapubic and retropubic drain is kept. Irrigation is started as a precautionary measure and wound is closed in layers. Traction is removed after 24 to 48 h and balloon deflated gradually. Foley's is removed on fifth and seventh post operative day and the patient is discharged after suture removal on tenth day.

Calculation of blood loss: Study of intraoperative blood loss was calculated by weight of sponges pre and post operatively soaked with blood. This gives the amount of loss in the sponge (1 gm = 1 ml)[3] to which the amount of blood in the suction bottle was added. Study of post operative blood loss was done by collecting the urine for 24 h and calculating the urine hemoglobin by photoelectric calorimeter by sinemethhaemoglobin method. Post operative blood loss was calculated by the formula:[4]

$\text{Post operative blood loss }=\frac{\text{Hb }\%\text{ Urine}}{\text{Hb}\%\text{ Patient}}\times \text{ volume of urine (24 h)}$

## RESULTS

The blood loss was calculated by the above formula for all the patients. Mean amount of postoperative blood loss was 18.9 ml. All the patients had blood loss in the range of 0 to 50 ml [Table 1]. None of the patient had blood loss greater than 50 ml.

**Table 1:** Blood loss

Blood loss in cc	Study group
0-50 cc	170
>50 cc	-
Average blood loss	18.9 ml

## DISCUSSION

McGill and Belfield described Suprapubic transvesical partial enucleation of the prostate in the late 1800s. Fuller and Frayer popularized the technique of complete enucleation of the gland.[5] However, hemostasis – in this procedure was far from satisfactory as the bleeders were not directly visible. The concept of control of postoperative hemorrhage by separation of the bladder neck from the prostate fossa was presented by Lower and Harris using non-absorbable bladder neck suture.[5] Hryntschak modified and popularized this technique in 1951. Dela Pena and Alcina proposed separation of the bladder cavity from the prostate fossa using a removable purse-string suture in 1962.[5] Malement popularized the removable partition suture, which is recommended only in cases of excessive bleeding in textbooks.[6–7]

The average blood loss in our study was 18.9 ml. All the patients in the study group had blood loss ranging from 10 to 50 ml. In our study, two patients required blood transfusion as the hemoglobin was less than 9 gm% preoperatively. *Naninga and O'Coner* in their study noted blood loss of more than 100ml in just 15% patients treated with balloon pressure traction technique which is little more in comparison with our study.[8]

Condie *et al*, reported 1% blood transfusion rate which is comparable with our study.[9] *Sheen and Quinlan* used early suture control at 3'o clock and 9'o clock for achieving hemostasis. They reported a mean blood loss of 841 ml in their study.[10] This is significantly higher than our study. This shows that Foley's pressure traction technique achieves better hemostasis than direct suture control. Similarly, *Moon* reported a blood transfusion rate of 83.3% in patients undergoing Freyer's Prostatectomy when standard technique was used.[11] This is significantly higher than our study [Table 2].

**Table 2:** Comparison of blood loss

Study group	Blood loss (ml)
Sheen and Quinlan[10]	841
Present study	18.9

*Ceylan K* reported a significant blood loss rate of only 3.2% in his study. He had used both sutures at 3'o clock and 9'o clock position as well as traction at the bladder neck to achieve hemostasis. As a corollary, it can thus be inferred that while suture technique alone is ineffective in achieving hemostasis, traction at the bladder neck achieves hemostasis.[12]

Traction at the bladder neck also has the advantage of allowing normal involution of the prostatic fossa and thus aids in ensuring complete hemostasis which is a significant advantage over the technique of keeping inflated balloon in the prostatic fossa, wherein this normal involution is hampered and rebound hemorrhage is common. *Goodyear and Beard* have shown by means of serial postoperative urethrogram that the prostatic fossa, like the uterus, contracts to 50% of its size within few minutes, to 25% in 6 to 12 hours and gradually it contracts completely. This is an important mechanism for achieving hemostasis postoperatively.[13]

In contrast to packing of prostatic fossa which interferes with this normal involutory response, the Foley's balloon pressure traction technique not only allows the normal contraction of prostatic fossa but rather aids it.

## CONCLUSION

The balloon pressure traction technique is an effective method of achieving haemostasis and avoids blood transfusion in almost all of the patients thus treated. Hence, open prostatectomy with Foley's balloon pressure traction technique for BPH is an acceptable option with a high degree of safety and efficacy in areas where the TUR-P equipment or the surgical expertise is lacking and out of reach of rural population.

## References

[1] Presti JC, Tanagho EA, McAnnich JW (2000). Neoplasms of the prostate gland. Smith's general Urology.

[2] Mearini E, Marzi M, Mearini L, Zucchi A, Porena M (1998). Open prostatectomy in benign prostatic hyperplazi: 10-Year experience in Italy. Eur Urol.

[3] David E, Neal, Kelly JD, Russell RC, Williams NS, Bulstrode CJ (2004). The Prostate and Seminal Vesicle. Bailey and love short practice of Surgery.

[4] Bruce AW, Zorab J, Still B (1960). Blood loss in Prostatic surgery. Br J Urol.

[5] Stutzman RE, Walsh PC, Walsh PC, Retik AB, Stamey TA, Vaughan ED (1992). Suprapubic and retropubik prostatectomy.

[6] Meier DE, Tarpley JL, Obioha O (1995). The outcome of suprapubic prostatectomy: A contemporary series in the developing world. Urology.

[7] Han M, Alfort HJ, Partin AW, Walsh PC, Retik AB, Vaughan ED, Wein AJ (2002). Retropubic and suprapubic open prostatectomy. Campbell's Urology.

[8] Naninga JB, Vicent J, O'Conor (1972). Suprapubic prostactomy: A review. J Urol.

[9] Condie JD, Cutherell L, Mian A (1999). Suprapubic prostatecyomy for benign prostatic hyperplasia in rural Asia: 200 Consecutive cases. Urology.

[10] Shaheen A, Quinlan D (2004). Feasibility of open simple prostatectomy with early vascular control. BJU Int.

[11] Moon HJ, Kim CK, Yoon JB (1970). Clinical study of prostatectomy. Korean J Urol.

[12] Ceylan K (2006). Open prostatectomy: The Results of a series of 320 cases in rural area. Eur J Med.

[13] Goodyear WA, Beard DE (1949). Blood loss in prostatectomy. J Urol.

